# Supplementary material for: Clinical Proof-of-Concept of a Non-Gene Editing Technology Using miRNA-Based shRNA to Engineer Allogeneic CAR T-Cells
Source: Int J Mol Sci. 2025 Feb 15;26(4):1658. doi: 10.3390/ijms26041658 (PMC11855736; doi:10.3390/ijms26041658)
Supplement: Supplementary file 1 [file ijms-26-01658-s001.zip › ijms-3443230-supplementary.pdf]

Article

Clinical Proof-of-Concept of a Non-Gene Editing Technology Using miRNA-Based shRNA to Engineer Allogeneic CAR T-Cells

Supplemental data

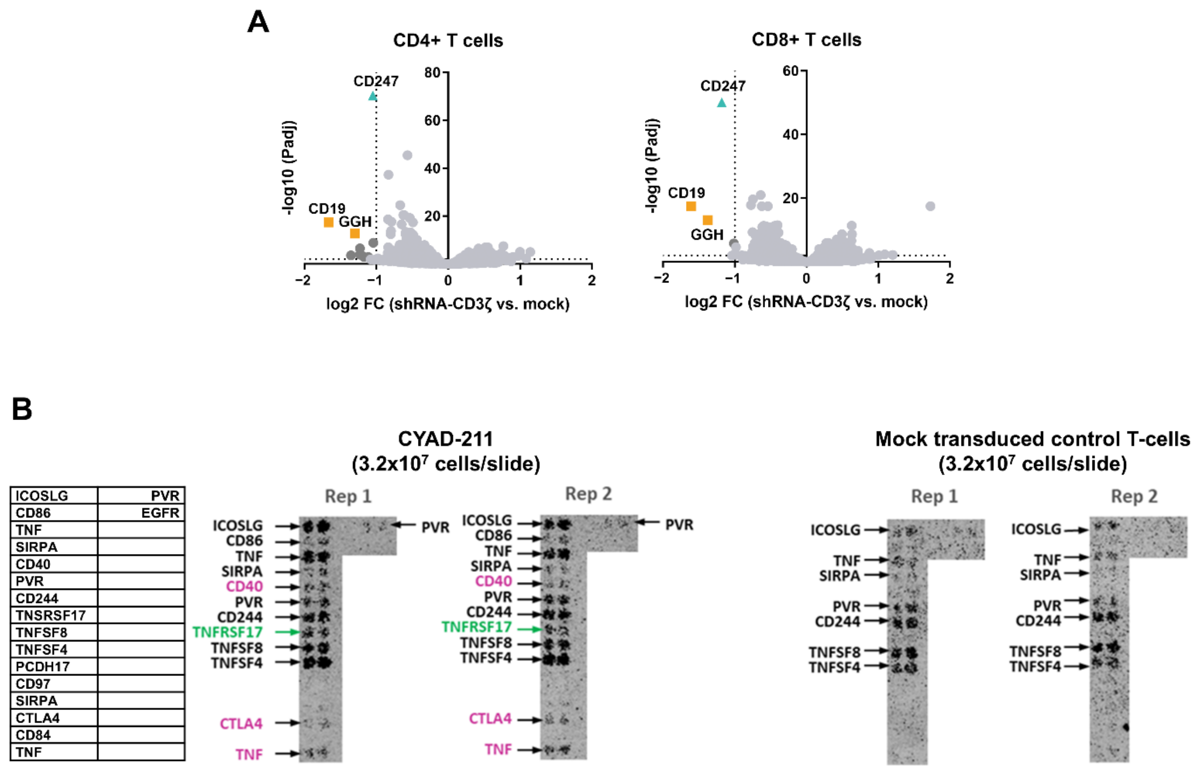

Figure S1 shRNA and anti-BCMA scFv specificity

A. Off target effects of the miRNA-based shRNA against CD3ζ. Volcano plots for differentially expressed genes in CD4+ and CD8+ T cells. For both statistical significance and fold change, predefined thresholds apply: a negative log10 (padj) above 2 (= padj < 0.01) and a log2 fold change (FC) below -1, respectively. All dots represent genes with a mean normalized count above 10. Dark grey dots are genes that exceed the threshold for statistical significance and fold change in expression; with yellow (CD19 and GGH) and green (CD247) dots meeting both criteria in both CD4+ and CD8+ populations. **B.** Assessment of the binding profile of CYAD-211 using a human plasma membrane protein cell array. First screening of the biding of BCMA-specific CAR T cells to HEK293 cells, individually expressing 5528 human proteins, comprising of cell surface membrane proteins and cell surface-tethered secreted proteins, identified seventeen primary hits. As a subsequent confirmation/specificity screen (shown here), the seventeen primary hits and one negative control receptor (EGFR) were over-expressed in HEK293 cells. Slides of fixed untransfected HEK293 cells (areas outside the spots) and HEK293 cells over-expressing all hits from the primary screens plus one positive control (spotted areas), were incubated with fluorescently-labelled test CYAD-211 cells (left) and Mock-transduced control T-cells (right) (n=2 slides per treatment). The CAR-specific T cell interaction is shown in green; the CAR-independent T-cell interactions are shown in black; and interactions that are not CAR-specific but have a large intensity difference are highlighted in pink (all known to be CAR-independent T-cell interactors and seen in previous Retrogenix T-cell screens). As expected, the only CAR-specific target was the primary target, BCMA (TNFRSF17). Background binding of the CAR T-cells to untransfected HEK293 cells was negligible.

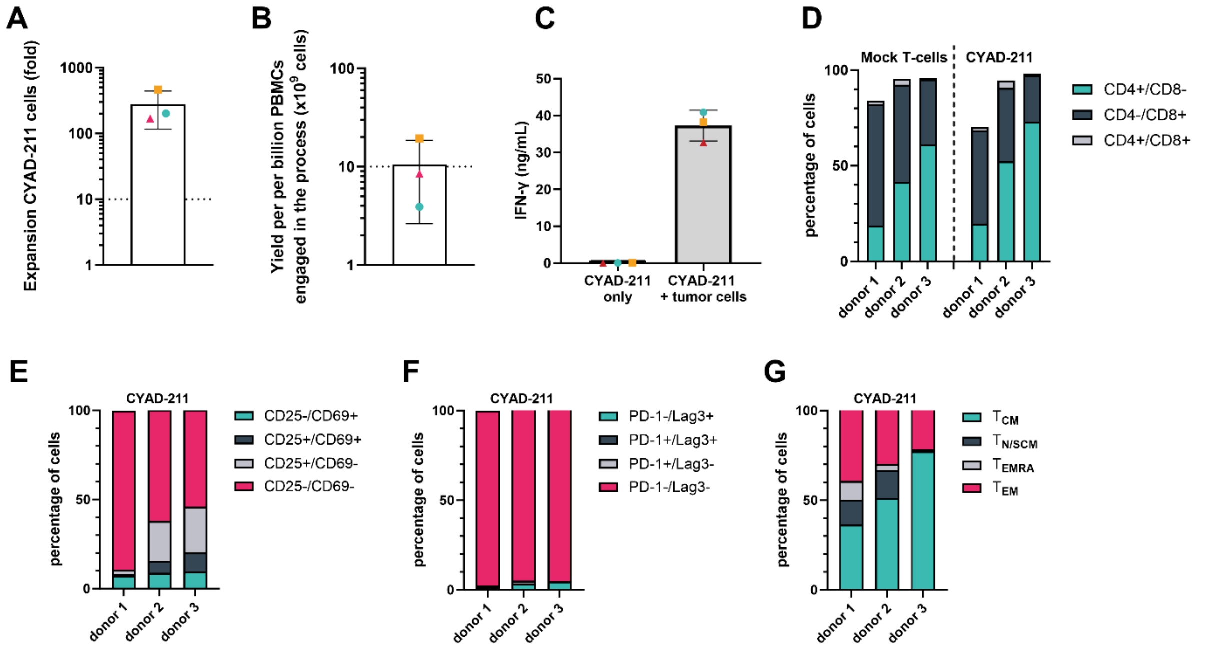

Figure S2 Characterization of the CYAD-211 clinical product

A and B. Transduced cell expansion and cell yield during CYAD-211 manufacturing illustrated by (A) the fold increase over the T-cells present in the apheresis and (B) the total number of CYAD-211 (in billions) generated from 1 billion of PBMCs engaged in the process. **C.** IFN-γ secretion in the supernatant of 24-hour 1:1 ratio co-cultures of CYAD-211 generated from 3 different healthy donors with BCMA-expressing RPMI-8226 cells, as measured by ELISA. **D.** CD4 or CD8 T-cell subtype proportions in the CYAD-211 product. **E**. T-cell activated phenotype characterization of the CYAD-211 product. **G**. T-cell activated exhaustion characterization of the CYAD-211 product. **F.** T-cell memory phenotype characterization of the CYAD-211 product. TCM: Central Memory T-cells (CD45RA−/CD62L+); TN/SCM: Naïve and Stem Cell Memory T-cells (CD45RA+/CD62L+), TEMRA: Effector memory (CD45RA+/CD62L−), TEM: Effector memory (CD45RA−/CD62L−). Early differentiated cells regroup both TCM and TN/SCM phenotypes.

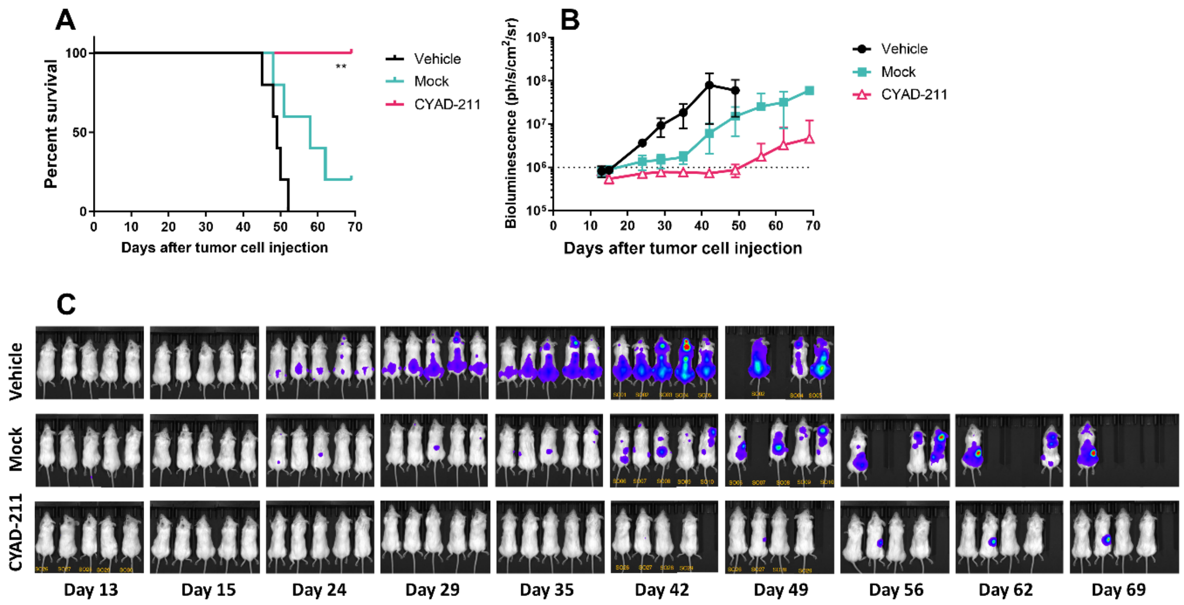

Figure S3 In vivo evidence of anti-tumor activity of CYAD-211.

NSG mice (n= 5 per group) were injected intravenously with vehicle or 10^7 CYAD-211 or 10^7 control T-cells from the same donor, 6 days following intravenous injection of $5x10^6$ RPMI-8226 multiple myeloma cancer cells. Control T-cells are cells transduced with the same vector backbone as CYAD-211, without the CAR or the shRNA. (A) Kaplan-Meier survival curves, (B) kinetics of bioluminescence emitted by luciferase-expressing KMS11-luc tumor cells), and (C) bioluminescence images of individual mice. One mouse in the group injected with CYAD-211 died on Day 35 following the bioluminescence acquisition and associated anesthesia. This mouse is not included in the Kaplan-Meier survival analysis. ** p value of < 0.01 (versus Mock)

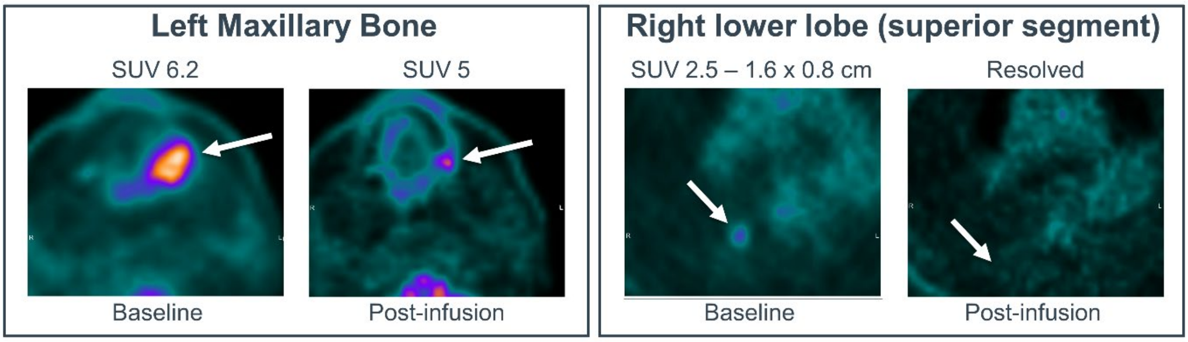

Figure S4 Response in individual lesions for patient #05

Computed Tomography Positron emission tomography (CT PET) scans of left maxillary bone and right lower lobe (superior segment) at baseline (1 month prior to infusion) and post CYAD-211 infusion (at Day 50) for patient #05.

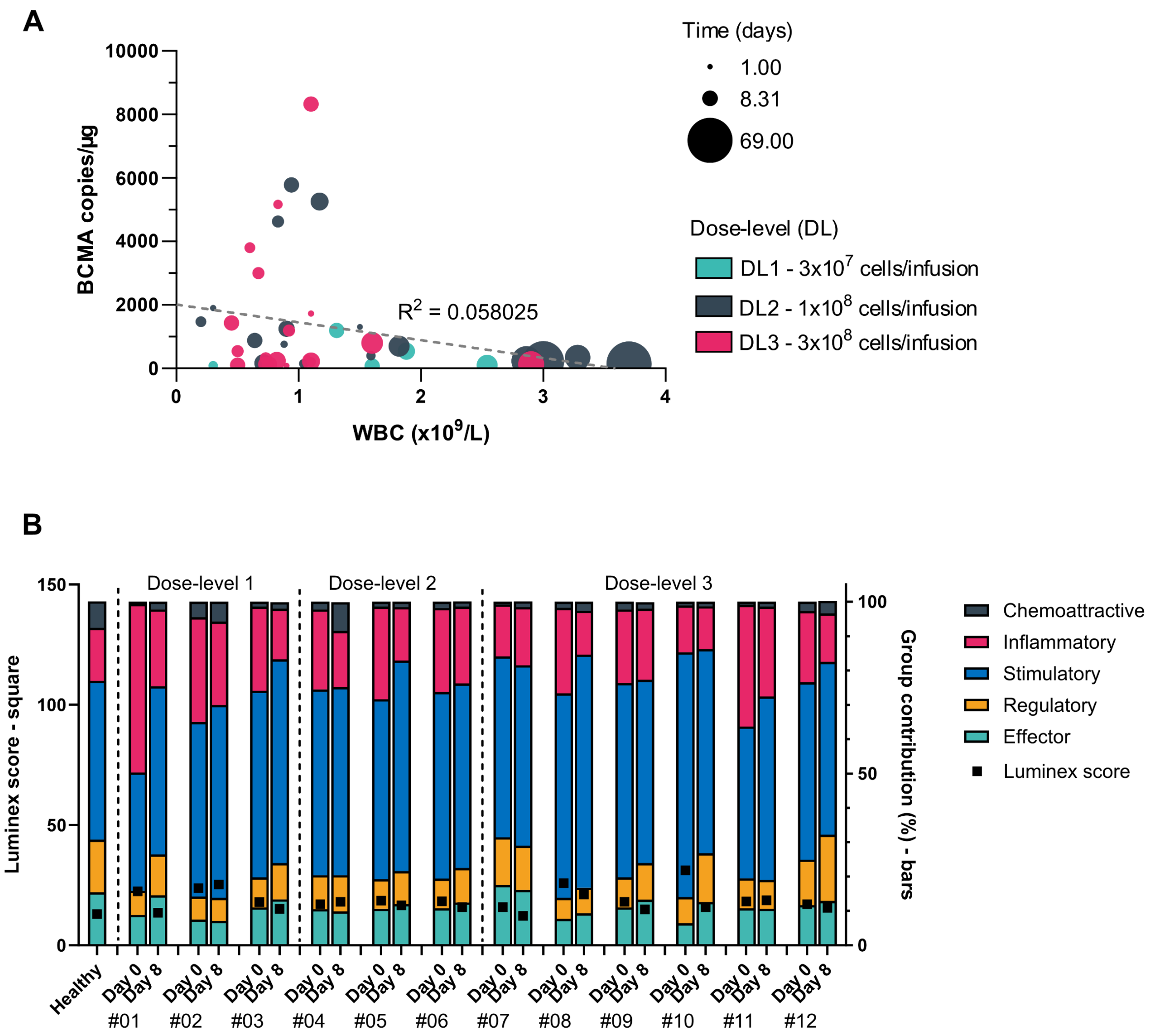

Figure S5 **Representation of the interactions between CYAD-211 cell kinetics and absolute white blood cells (WBC) kinetics and cytokine modulation post CYAD-211 infusion**

A. Each dot represents the value of CYAD-211 copies per µg of blood, the value of absolute WBC count, at a specific timepoint and for each specific patient. CYAD-211 kinetics was determined by digital droplet polymerase chain reaction on genomic DNA from peripheral blood mononuclear cells isolated from blood collected at pre-specified timepoints. Values below the lower limit of quantification (LLOQ) are not shown. Data were pooled per dose-level (DL). The dotted line represents the 4th order polynomial regression curve fitting data approximation. **B.** Serum cytokine modulation as measured by Luminex immunoassay at baseline (Day 0 before CYAD-211 infusion) and after CYAD-211 infusion (Day 8). Cytokines were grouped across 5 functional groups and Luminex score and group contributions were calculated for both timepoint. Luminex score and functional group contribution were compared to those of five healthy donors.

	HLA locus	A		B		C		DRB1		DQB1		DPB1		Anti-donor HLA antibodies
	**allele**	**#1**	**#2**	**#1**	**#2**	**#1**	**#2**	**#1**	**#2**	**#1**	**#2**	**#1**	**#2**	
	**Donor**	**02:01**	**03:01**	**39:06:02G**	**44:02**	**05:01**	**07:02**	**01:01**	**15:01**	**05:01**	**06:02**	**03:01**	**04:01**	/
**Patient**	**#01**	01:01	03:01	35:01	57:01	04:01	06:02	01:01	11:01	03:01	05:01	04:01	04:01	None
	**#02**	02:01	33:03	14:02	51:01	08:02	14:02	03:01	07:01	02:01	02:01	04:01	10:01	None
	**#03**	02:01	02:01	27:05	40:01	02:02	03:04	13:02	15:01	06:04	06:02	02:01	10:01	None
	**#04**	02:01	24:02	27:05	35:02	04:01	07:04	11:04	11:01	03:01	03:01	04:01	04:01	None
	**#05**	24:02	31:01	40:01	44:05	02:02	03:04	04:04	16:01	03:02	05:02	02:01	06:01	None
	**#06**	02:01	31:01	07:02	15:20	07:02	04:01	13:01	08:02	06:03	04:02	04:02	10:01	None
	**#07**	03:01	11:01	27:05	37:01	01:02	06:02	08:01	11:01	03:01	04:02	04:02	04:02	None
	**#08**	02:01	03:01	18:05	51:01	01:02	12:03	13:02	14:01	05:03	06:04	02:01	04:01	None
	**#09**	03:01	03:01	07:02	07:02	07:02	07:02	15:01	15:01	06:02	06:02	04:01	04:01	None
	**#10**	03:01	68:01	07:02	40:01	03:04	07:02	13:02	15:01	06:04	06:02	03:01	04:01	None
	**#11**	02:01	24:02	18:01	35:02	07:01	04:01	11:04	14:54	03:01	05:03	04:02	02:01	None
	**#12**	24:02	24:02	35:03	44:03	02:02	04:54	07:01	11:01	02:01	03:01	02:01	04:02	None

Table S1 Summary of anti-HLA antibodies detected in patients post CYAD-211 infusion.
Anti-HLA Type I or II antibodies were evaluated in patient's samples at different timepoints by solid phase single antigen bead assay from Immucor. Data showed very low prevalence of pre-existing anti-HLA antibodies and no patient had detectable anti-donor HLA antibodies. HLA genotyping of the donor and patients was done by next-generation sequencing [NGS] up to 6 digits. Full match with the donor's HLA genotype (4 digits) for a specific allele is shown in green, partial match (2 digits) is shown in grey.

Supplemental Material and Methods

Sequence of the BCMA-targeting scFv

ATGGCTTTGCCAGTGACAGCACTGCTCTTGCCCCTCGCACTCCTCCTCCACGCAGCTCGC
CCCCAGCTCCAATTGCAGGAAAGCGGTCCGGGGCTTGTTAAACCTTCCGAGACTCTGTCACTG
ACTTGCACAGTCTCTGGTGGAAGTATCAGTTCTGGCAGTTACTTCTGGGGTTGGATAAGGCAA
CCTCCTGGTAAGGGTTTGGAGTGGATCGGGTCTATTTATTACTCCGGCATCACCTACTACAAC
CCAAGCCTGAAATCTCGAGTCACCATCTCTGTCGACACTTCCAAAAACCAGTTTAGCCTCAAA
CTTTCCAGCGTTACAGCAGCAGATACTGCCGTCTATTACTGTGCCCGACACGACGGTGCAGTA
GCCGGCCTGTTCGATTACTGGGGTCAGGGAACCCTTGTTACTGTTAGCAGCGGCGGAGGGGG
ATCCGGAGGTGGAGGATCAGGGGGAGGTGGATCCTATGTTCTTACACAACCACCTTCTGTGA
GCGTTGCACCTGGTCAAACCGCACGAATCACCTGTGGCGGGAATAATATTGGGTCTAAAAGC
GTTCACTGGTATCAGCAGCCGCCTGGCCAAGCACCGGTCGTGGTTGTTTATGATGATTCTGAT
CGGCCATCCGGGATCCCCGAACGGTTCAGTGGCAGTAACTCTGGGAATACAGCAACTCTCAC
CATATCACGGGTTGAAGCTGGCGACGAAGCCGTTTATTACTGCCAGGTATGGGATAGTAGTA
GTGATCATGTTGTATTCGGGGGCGGCACCAAACTCACCGTTTTGAGTAGT

Sequence of the BCMA-specific CAR-tCD34-CD3ζ shRNA construct

AAGCTTTGCTCTTAGGAGTTTCCTAATACATCCCAAACTCAAATATATAAAGCATTTGAC
TTGTTCTATGCCCTAGGGGGCGGGGGGAAGCTAAGCCAGCTTTTTTTAACATTTAAAATGTTA
ATTCCATTTTAAATGCACAGATGTTTTTATTTCATAAGGGTTTCAATGTGCATGAATGCTGCAA
TATTCCTGTTACCAAAGCTAGTATAAATAAAAATAGATAAACGTGGAAATTACTTAGAGTTTC
TGTCATTAACGTTTCCTTCCTCAGTTGACAACATAAATGCGCTGCTGAGAAGCCAGTTTGCAT
CTGTCAGGATCAATTTCCCATTATGCCAGTCATATTAATTACTAGTCAATTAGTTGATTTTTATT
TTTGACATATACATGTGAAAGACCCCACCTGTAGGTTTGGCAAGCTAGCTTAAGTAACGCCAT
TTTGCAAGGCATGGAAAAATACATAACTGAGAATAGAAAAGTTCAGATCAAGGTCAGGAAC
AGATGGAACAGCTGAATATGGGCCAAACAGGATATCTGTGGTAAGCAGTTCCTGCCCCGGCT
CAGGGCCAAGAACAGATGGAACAGCTGAATATGGGCCAAACAGGATATCTGTGGTAAGCAG
TTCCTGCCCCGGCTCAGGGCCAAGAACAGATGGTCCCCAGATGCGGTCCAGCCCTCAGCAGT
TTCTAGAGAACCATCAGATGTTTCCAGGGTGCCCCAAGGACCTGAAATGACCCTGTGCCTTAT
TTGAACTAACCAATCAGTTCGCTTCTCGCTTCTGTTCGCGCGCTTCTGCTCCCCGAGCTCAATA
AAAGAGCCCACAACCCCTCACTCGGCGCGCCAGTCCTCCGATTGACTGAGTCGCCCGGGTAC
CCGTGTATCCAATAAACCCTCTTGCAGTTGCATCCGACTTGTGGTCTCGCTGTTCCTTGGGAGG
GTCTCCTCTGAGTGATTGACTACCCGTCAGCGGGGGTCTTTCATTTGGGGGCTCGTCCGGGAT
CGGGAGACCCCTGCCCAGGGACCACCGACCCACCACCGGGAGGTAAGCTGGCCAGCAACTT
ATCTGTGTCTGTCCGATTGTCTAGTGTCTATGACTGATTTTATGCGCCTGCGTCGGTACTAGTT
AGCTAACTAGCTCTGTATCTGGCGGACCCGTGGTGGAACTGACGAGTTCGGAACACCCGGCC
GCAACCCTGGGAGACGTCCCAGGGACTTCGGGGGCCGTTTTTGTGGCCCGACCTGAGTCCTA
AAATCCCGATCGTTTAGGACTCTTTGGTGCACCCCCCTTAGAGGAGGGATATGTGGTTCTGGT
AGGAGACGAGAACCTAAAACAGTTCCCGCCTCCGTCTGAATTTTTGCTTTCGGTTTGGGACCG
AAGCCGCGCCGCGCGTCTTGTCTGCTGCAGCATCGTTCTGTGTTGTCTCTGTCTGACTGTGTTT
CTGTATTTGTCTGAAAATATGGGCCCGGGCTAGCCTGTTACCACTCCCTTAAGTTTGACCTTAG
GTCACTGGAAAGATGTCGAGCGGATCGCTCACAACCAGTCGGTAGATGTCAAGAAGAGACG
TTGGGTTACCTTCTGCTCTGCAGAATGGCCAACCTTTAACGTCGGATGGCCGCGAGACGGCAC
CTTTAACCGAGACCTCATCACCCAGGTTAAGATCAAGGTCTTTTCACCTGGCCCGCATGGACA
CCCAGACCAGGTCCCCTACATCGTGACCTGGGAAGCCTTGGCTTTTGACCCCCCTCCCTGGGT
CAAGCCCTTTGTACACCCTAAGCCTCCGCCTCCTCTTCCTCCATCCGCCCCGTCTCTCCCCCTT
GAACCTCCTCGTTCGACCCCGCCTCGATCCTCCCTTTATCCAGCCCTCACTCCTTCTCTAGGCG
CCCCCATATGGCCATATGAGATCTTATATGGGGCACCCCCGCCCCTTGTAAACTTCCCTGACC
CTGACATGACAAGAGTTACTAACAGCCCCTCTCTCCAAGCTCACTTACAGGCTCTCTACTTAG

TCCAGCACGAAGTCTGGAGACCTCTGGCGGCAGCCTACCAAGAACAACTGGACCGACCGGT
GGTACCTCACCCTTACCGAGTCGGCGACACAGTGTGGGTCCGCCGACACCAGACTAAGAACC
TAGAACCTCGCTGGAAAGGACCTTACACAGTCCTGCTGACCACCCCCACCGCCCTCAAAGTA
GACGGCATCGCAGCTTGGATACACGCCGCCCACGTGAAGGCTGCCGACCCCGGGGGTGGAC
CATCCTCTAGACTGCCATGGATGGCTTTGCCAGTGACAGCACTGCTCTTGCCCCTCGCACTCC
TCCTCCACGCAGCTCGCCCCCAGCTCCAATTGCAGGAAAGCGGTCCGGGGCTTGTTAAACCTT
CCGAGACTCTGTCACTGACTTGCACAGTCTCTGGTGGAAGTATCAGTTCTGGCAGTTACTTCT
GGGGTTGGATAAGGCAACCTCCTGGTAAGGGTTTGGAGTGGATCGGGTCTATTTATTACTCCG
GCATCACCTACTACAACCCAAGCCTGAAATCTCGAGTCACCATCTCTGTCGACACTTCCAAA
AACCAGTTTAGCCTCAAACTTTCCAGCGTTACAGCAGCAGATACTGCCGTCTATTACTGTGCC
CGACACGACGGTGCAGTAGCCGGCCTGTTCGATTACTGGGGTCAGGGAACCCTTGTTACTGTT
AGCAGCGGCGGAGGGGGATCCGGAGGTGGAGGATCAGGGGGAGGTGGATCCTATGTTCTTA
CACAACCACCTTCTGTGAGCGTTGCACCTGGTCAAACCGCACGAATCACCTGTGGCGGGAAT
AATATTGGGTCTAAAAGCGTTCACTGGTATCAGCAGCCGCCTGGCCAAGCACCGGTCGTGGT
TGTTTATGATGATTCTGATCGGCCATCCGGGATTCCCGAACGGTTCAGTGGCAGTAACTCTGG
GAATACAGCAACTCTCACCATATCACGGGTTGAAGCTGGCGACGAAGCCGTTTATTACTGCC
AGGTATGGGATAGTAGTAGTGATCATGTTGTATTCGGGGGCGGCACCAAACTCACCGTTTTGA
GTAGTGCGGCCGCGTTCGTGCCGGTCTTCCTGCCAGCGAAGCCCACCACGACGCCAGCGCCG
CGACCACCAACACCGGCGCCCACCATCGCGTCGCAGCCCCTGTCCCTGCGCCCAGAGGCGTG
CCGGCCAGCGGCGGGGGGCGCAGTGCACACGAGGGGGCTGGACTTCGCCTGTGATATCTAC
ATCTGGGCGCCCCTGGCCGGGACTTGTGGGGTCCTTCTCCTGTCACTGGTTATCACCCTTTACT
GCAACCACAGGAACAAACGGGGCAGAAAGAAACTCCTGTATATATTCAAACAACCATTTAT
GAGACCAGTACAAACTACTCAAGAGGAAGATGGCTGTAGCTGCCGATTTCCAGAAGAAGAA
GAAGGAGGATGTGAACTGAGAGTGAAGTTCAGCAGGAGCGCAGACGCCCCCGCGTACCAGC
AGGGCCAGAACCAGCTCTATAACGAGCTCAATCTAGGACGAAGAGAGGAGTACGATGTTTT
GGACAAGAGACGTGGCCGGGACCCTGAGATGGGGGGAAAGCCGCAGAGAAGGAAGAACCC
TCAGGAAGGCCTGTACAATGAACTGCAGAAAGATAAGATGGCGGAGGCCTACAGTGAGATT
GGGATGAAAGGCGAGCGCCGGAGGGGCAAGGGGCACGATGGCCTTTACCAGGGTCTCAGTA
CAGCCACCAAGGACACCTACGACGCCCTTCACATGCAGGCCCTGCCCCCTCGCGCCCGCGCC
AAAAGGTCTGGCTCCGGTGAGGGCAGAGGAAGTCTTATAACATGCGGTGACGTGGAGGAGA
ATCCCGGCCCTATGCCTCGCGGCTGGACAGCCCTGTGCCTGCTGTCTCTGCTGCCATCCGGCTT
CATGAGCCTGGATAATAACGGCACAGCCACCCCAGAGCTGCCTACACAGGGCACCTTCAGC
AATGTGTCCACAAACGTGAGCTATCAGGAGACCACAACCCCTTCTACCCTGGGATCCACAAG
CCTGCACCCCGTGTCTCAGCACGGCAACGAAGCCACCACCAACATCACCGAGACCACAGTG
AAGTTTACCTCCACCTCTGTGATTACCTCTGTGTACGGAAATACAAACTCCAGCGTGCAGTCT
CAGACATCTGTGATCTCCACAGTGTTTACAACACCTGCCAATGTGTCCACCCCAGAGACAAC
CCTGAAGCCCAGCCTGTCTCCTGGAAATGTGTCCGATCTGTCTACCACCTCCACCAGCCTGGC
CACCTCTCCCACCAAGCCCTATACCTCCTCTTCTCCCATCCTGAGCGATATCAAAGCCGAGAT
CAAATGCAGCGGGATTCGGGAAGTGAAACTGACACAGGGCATCTGCCTGGAACAGAATAAG
ACATCCAGCTGCGCCGAGTTTAAGAAAGATAGAGGAGAGGGACTGGCCAGGGTGCTGTGTG
GCGAAGAGCAGGCCGACGCCGATGCCGGCGCCCAGGTGTGTTCCCTGCTGCTGGCCCAGTCT
GAGGTGCGCCCCCAGTGCCTGCTGCTGGTGCTGGCCAATCGGACAGAAATTAGCAGCAAGCT
GCAGCTGATGAAAAAACACCAGAGCGATCTGAAAAAGCTGGGCATCCTGGACTTTACCGAG
CAGGACGTGGCCTCTCACCAGAGCTACAGCCAGAAAACACTGATCGCCCTGGTGACCAGCG
GAGCCCTGCTGGCCGTGCTGGGCATCACCGGATATTTCCTGATGAATAGGCGCAGCTGGAGC
CCCACCGGCGAGCGGCTGGAGCTGGAGCCTTAACTCGAGAGATCTAAAGGGGGACGGGGCT
GAATTTCTTCCTTCCCCAACCCCCTTCCCTTCTCCTCCAGATAGATGCAAAGCTGAATCTCCCG
CCCTGCTCGCTCAGCTGATCTGTGGCTTAATCTGGGCGTCTGCAGGGGGATTGAGTTTTGAAC

TCCCTGCAGTCGCCCAGATTGAGTTACATCAGTCGGTTTTCGTCGAGGGCCCCAATCGAGCTC
TCCCACTCCTACCCTCCCCAGTGGGACTGCCCCACTGCCCCCTCCCAGATAGG

Sequence of the three shRNA guide sequences targeting CD3ζ

- shRNA CD3ζ-1 guide strand: 5′_AAGCCACTCAGACACAGAG_3′
- shRNA CD3ζ-2 guide strand: 5′_TAATCTGGGCGTCTGCAGG_3
- shRNA CD3ζ-3 guide strand: 5′_TAATGACGCAGCAGTATCC_3′

Sequence of the tCD19-CD3ζ shRNA construct

ATGCCACCTCCTCGCCTCCTCTTCTTCCTCCTCTTCCTCACCCCCATGGAAGTCAGGCCCG
AGGAACCTCTAGTGGTGAAGGTGGAAGAGGGAGATAACGCTGTGCTGCAGTGCCTCAAGGG
GACCTCAGATGGCCCCACTCAGCAGCTGACCTGGTCTCGGGAGTCCCCGCTTAAACCCTTCTT
AAAACTCAGCCTGGGGCTGCCAGGCCTGGGAATCCACATGAGGCCCCTGGCCATCTGGCTTT
TCATCTTCAACGTCTCTCAACAGATGGGGGGCTTCTACCTGTGCCAGCCGGGGCCCCCCTCTG
AGAAGGCCTGGCAGCCTGGCTGGACAGTCAATGTGGAGGGCAGCGGGGAGCTGTTCCGGTG
GAATGTTTCGGACCTAGGTGGCCTGGGCTGTGGCCTGAAGAACAGGTCCTCAGAGGGCCCCA
GCTCCCCTTCCGGGAAGCTCATGAGCCCCAAGCTGTATGTGTGGGCCAAAGACCGCCCTGAG
ATCTGGGAGGGAGAGCCTCCGTGTCTCCCACCGAGGGACAGCCTGAACCAGAGCCTCAGCC
AGGACCTCACCATGGCCCCTGGCTCCACACTCTGGCTGTCCTGTGGGGTACCCCCTGACTCTG
TGTCCAGGGGCCCCCTCTCCTGGACCCATGTGCACCCCAAGGGGCCTAAGTCATTGCTGAGCC
TAGAGCTGAAGGACGATCGCCCGGCCAGAGATATGTGGGTAATGGAGACGGGTCTGTTGTTG
CCCCGGGCCACAGCTCAAGACGCTGGAAAGTATTATTGTCACCGTGGCAACCTGACCATGTC
ATTCCACCTGGAGATCACTGCTCGGCCAGTACTATGGCACTGGCTGCTGAGGACTGGTGGCTG
GAAGGTCTCAGCTGTGACTTTGGCTTATCTGATCTTCTGCCTGTGTTCCCTTGTGGGCATTCTTC
ATCTTCAAAGAGCCCTGGTCCTGAGGAGGAAAAGAAAGCGAATGACTTAACATGGTCAACG
CGTTAGCATGCACCTCGAGAGATCTAAAGGGGGACGGGGCTGAATTTCTTCCTTCCCCAACC
CCCTTCCCTTCTCCTCCAGATAGATGCAAAGCTGAATCTCCCGCCCTGCTCGCTCAGCTGATCT
GTGGCTTAATCTGGGCGTCTGCAGGGGGATTGAGTTTTGAACTCCCTGCAGTCGCCCAGATTG
AGTTACATCAGTCGGTTTTCGTCGAGGGCCCCAATCGAGCTCTCCCACTCCTACCCTCCCCAG
TGGGACTGCCCCACTGCCCCCTCCCAGATA

Evaluation of off-target effects of the miRNA-based construct targeting CD3ζ

In order to assess whether the expression of the miRNA-based construct targeting CD3ζ may result in unanticipated off-target effects at the transcriptome level, the transcriptional profiles of isolated CD4+ and CD8+ cells transduced with control or shRNA-CD3ζ encoding viral vectors from three healthy donors were analyzed. Differential analysis between control and shRNA-CD3ζ transduced T-cells revealed three genes (CD247, Gamma-Glutamyl Hydrolase (GGH) and CD19) whose expression was statistically significantly affected in both CD4+ and CD8+ T-cells. Normalized counts suggested low relative abundance of the CD19 and GGH mRNA of in both Mock and miRNA-based construct-transduced cells.

Binding profile of CYAD-211 using a human plasma membrane protein cell array

Microarray technology (performed by Retrogenix) was used to screen for specific off-target binding interactions of a CYAD-211. For the primary screening, 5528 expression vectors, each encoding a full-length human plasma membrane protein or a full-length cell surface-tethered human secreted protein, were arrayed in duplicate across 16 microarray slides (‘slide-sets’). Human HEK293 cells were used for reverse transfection/expression. The test CAR T-cells were fluorescently labelled with a FarRed fluorescent dye and added to each slide after cell fixation at a cell density of 3.2 x 10^7/slide. Fluorescent images were analyzed and quantitated (for transfection) using ImageQuant software. A protein ‘hit’ was defined as a duplicate spot showing a raised signal compared to background levels. This is achieved by visual inspection using the images gridded on the ImageQuant software. Hits were classified as ‘strong, medium, weak or very weak’, depending on the intensity of the duplicate spots.

To confirm the hits and assess specificity, vectors encoding all hits identified on one or both of the two replicate slides, plus a control vector encoding EGFR, were arrayed and expressed in HEK293 cells on new slides. Cells were then fixed. Identical slides were treated with fluorescently-labelled test CAR T-cells and fluorescently-labelled control T-cells (n=2 slides per treatment). Slides were imaged for fluorescence and analyzed like for the primary screen.

Serum cytokine analysis by Luminex technology

Systemic levels of 18 cytokines and chemokines were measured in serum isolated from whole blood from the 12 patients before the CYAD-211 infusion and at Day 8 post infusion, which corresponds to the peak of detection of CYAD-211 in the blood using ProcartaPlex Immunoassays (ThermoFisher Scientific) according to the manufacturers' specifications. Samples were acquired in duplicates and analytes concentration was determined relative to standards supplied by the manufacturer. Data acquisition and analysis were performed using a MAGPIX instrument (ThermoFisher Scientific) and ProcartaPlex data analysis software. The mean of the duplicates was calculated for each analyte. Values below the lower limit of quantification (LLOQ) were set at LLOQ, and values above the upper limit of quantification (ULOQ) were set at ULOQ. For each analyte, the mean value was normalized by a "healthy donor value" of the corresponding analyte (mean concentration of the analyte among five healthy donors). The Luminex score is the sum at each specific timepoint of the normalized value of the 18 analytes: interferon gamma (IFN-γ), tumor necrosis factor (TNF-α), interleukin (IL)-2, -4, -6, -7, -8, -10, -12p70, -15 and -13, granulocyte-macrophage colony-stimulating factor (GM-CSF), monocyte chemoattractant protein-1 (MCP-1), interferon gamma-induced protein 10 (IP-10) and RANTES. To give an informative overview of the results, analytes were pooled according to functional groups depending on their biological function, and the contribution of each group to the total Luminex score was calculated: effector (IFN-γ and TNF-α), regulatory (IL-4, -10 and -13), stimulatory (GM-CSF, IL-2, -7, -8, -12p70 and IL-15) inflammatory (IL-6 and MCP-1) and chemoattractive (IP-10 and RANTES).

Quantification of vector copy number (VCN)

Quantitative PCR (qPCR) for VCN was performed, in multiplex, on CYAD-211 cell genomic DNA using primers and labeled probes (Integrated DNA Technologies (IDT)) specific for the CAR construct. The amplification of a second gene (i.e. the housekeeping gene coding for human albumin) was used (i) as an internal control of amplification as well as (ii) for normalization. Copies of retroviral DNA per cell were estimated based by a comparison to linearized BCMA CAR and albumin plasmids mix of known ratio assayed in the same experiment.

Quantification of replication competent retrovirus (RCR)

The detection of RCR was assessed in CYAD-211 gDNA by a limit detection probe-based qPCR. Primers and probe were specifically designed to amplify the Gibbon Ape Leukemia Virus (GALV) envelope (env) gene. A second gene (i.e. the housekeeping gene coding for human albumin) was used (i) as an internal control of amplification as well as (ii) for normalization. A mix of Albumin and pUC57-GalV linearized plasmids was used as a positive control.
